# Factors related to the location of pigment epithelial detachment in central serous chorioretinopathy

**DOI:** 10.1038/s41598-022-08550-0

**Published:** 2022-03-16

**Authors:** Young Ho Kim, Edward Kang, Jaeryung Oh

**Affiliations:** Department of Ophthalmology, Korea University College of Medicine, 73 Goryeodae-ro Sungbuk-ku, Seoul, 02841 Korea

**Keywords:** Diseases, Medical research, Pathogenesis

## Abstract

Pigment epithelial detachment (PED) is common in eyes with central serous chorioretinopathy (CSC), and choroidal neovascularisation (CNV), which is almost always associated with PED, is found in a higher proportion than previously expected. Using *en-face* optical coherence tomography, this retrospective study aimed to investigate the PED location in relation to various geometric landmarks including the foveal centre (FC), greatest choroidal thickness (GCT) point and optic disc centre. In a total of 98 eyes, the distance from the FC to PED centroid was correlated with the ratio of GCT to subfoveal choroidal thickness (*r* = 0.278, *P* = 0.006) and the distance from the FC to GCT point (*r* = 0.371, *P* < 0.001). Eyes with CNV had a shorter distance between the PED centroid and FC (700 ± 439 μm) than those without (1191 ± 964 μm, *P* = 0.001). Analysis of covariance showed that the distance from the FC to the PED centroid was significantly correlated with the distance from the FC to the GCT point (*P* = 0.009) and the PED group with and without CNV (*P* = 0.020). This result suggests that the development of complicated PED with CNV can be related to both choroidal vascular abnormalities and retinal pigment epithelial insufficiency.

## Introduction

Central serous chorioretinopathy (CSC) is a disease characterized by serous retinal detachment and fluid accumulation in the subretinal space, usually involving the macular centre^1–3^. Serous retinal detachment in eyes with CSC results from the leakage of subretinal fluid (SRF) at the retinal pigment epithelium (RPE) level^3^. Typically, pigment epithelial detachment (PED) is observed within or adjacent to the serous retinal detachment and serves as an access pathway for serous fluid from the choriocapillaris to the subretinal space^1,4,5^. Many studies have shown that choroidal vascular abnormalities play a primary role in the pathogenesis of CSC^6,7^. Previous studies have reported that the location of the PED corresponds to the leaking point on fluorescein angiography (FA)^5,8–10^ and the choroidal hyperfluorescent region on indocyanine green angiography (ICGA)^10,11^. These findings suggest that the increased choroidal hydrostatic pressure caused by choroidal congestion and hyperpermeability is an important pathogenic process in the development of PED in CSC^12,13^.

With the advent of enhanced depth imaging optical coherence tomography (EDI-OCT) and swept-source OCT (SS-OCT), it has been suggested that localized ischemia of the choriocapillaris caused by mechanical compression of the underlying large choroidal vessel causes distress to the overlying RPE^6,14^. The dome-shape or flat irregular PEDs were found above the dilated large choroidal vessels and thickened choroid in CSC^15,16^. The relationship between large choroidal vessels and the development of complicated PED with choroidal neovascularisation (CNV) or persistent SRF has been widely investigated in previous studies using OCT and OCT angiography^15,17–19^. Recent advances in *en face* OCT technology have provided a method for determining the morphometric measurements of PED and SRF^4,15,20^. However, quantitative measurements of PED location and its relationship to choroidal geometry in CSC have not been widely evaluated.

In this study with SS-OCT, we hypothesized that the development of complicated PED by persistent SRF or CNV in CSC could be related to both choroidal and retinal geometries. And we investigated the factors influencing the location of PED using *en face* SS-OCT images.

## Methods

This study adhered to the tenets of the Declaration of Helsinki and was approved by the Institutional Review Board of Korea University Hospital, Seoul, Korea (IRB number: 2021AN0469). We retrospectively analysed the data of patients with CSC obtained from the OCT database between April 2015 and May 2021. We excluded eyes with retinal diseases other than CSC or a previous history of vitreoretinal surgery. Patients underwent a comprehensive examination, including fundus photography (FP), SS-OCT and OCT angiography (DRI OCT Triton, software version 10.17.003.01; Topcon Corp., Tokyo, Japan), fluorescein angiography (FA) and indocyanine green angiography (ICGA, Spectralis HRA2; Heidelberg Engineering, Heidelberg, Germany). CSC was diagnosed when serous retinal detachment involving the posterior pole and the area of RPE alteration were identified on SS-OCT, accompanied by fluorescein leakage at the level of the RPE on FA^21^. Subjects with poor image quality were excluded.

### Measurement of choroidal thickness on OCT image

**Figure 1:**
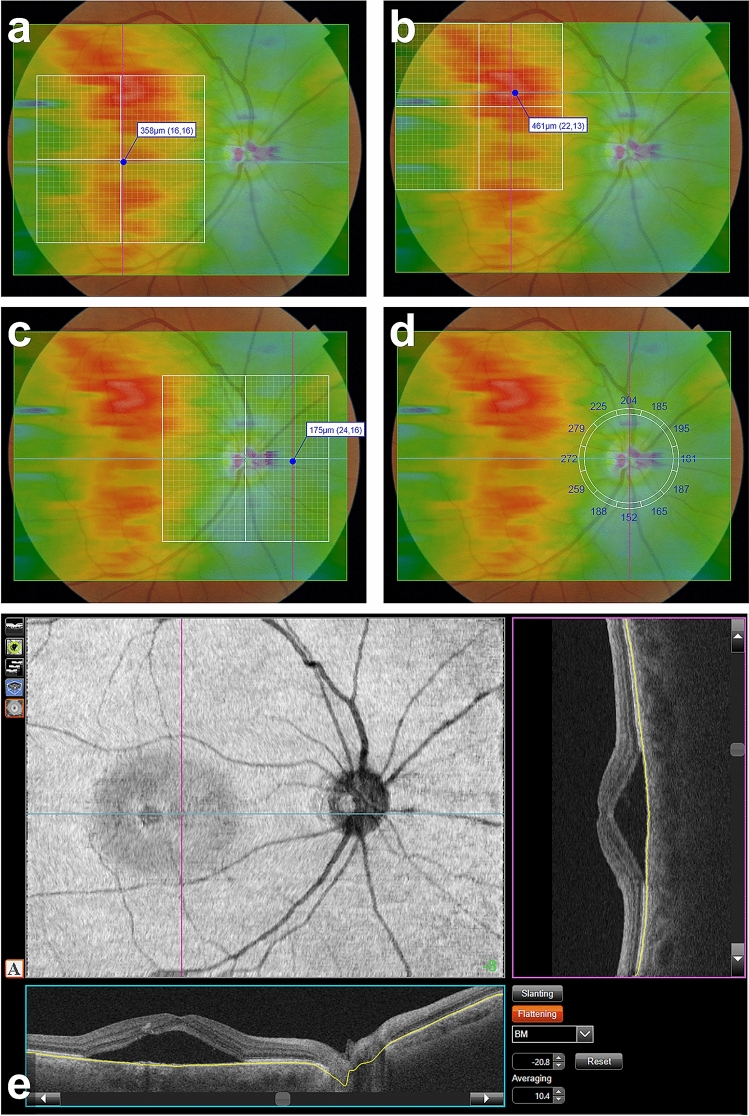
Measurement of choroidal thickness (CT) and determination of choroidal and retinal geometric landmarks. Each CT was measured using a color-coded CT map and a SuperPixel-200 grid on swept-source optical coherence tomography (SS-OCT). (**a**) Subfoveal choroidal thickness (SCT), (**b**) greatest choroidal thickness (GCT), and (**c**) nasal peripapillary choroidal thickness (NPCT) were measured semi-automatically after the superimposition of a color-coded CT map and a SuperPixel-200 grid on the color fundus photograph using the manufacturer’s software. (**d**) The location of NPCT measurement was determined using a 3.4-mm diameter circular grid for retinal nerve fiber layer analysis. The locations of the three reference geometric landmarks – the foveal centre, optic disc centre, and GCT point – were determined on en face SS-OCT images using the built-in software (**d** and **e**). The foveal centre and optic disc centre were marked on the en face image at the corresponding point where the two vertical lines intersected. The x and y coordinates of the GCT point was determined during the GCT measurement using a SuperPixel-200 grid (**b**).

The three parameters of choroidal thickness (CT), subfoveal CT (SCT), nasal peripapillary CT (NPCT), and greatest CT (GCT), were measured semi-automatically using a method modified from previously reported studies (Fig. 1)^22,23^. In brief, we measured CTs after superimposing a color-coded CT map and a SuperPixel-200 grid on the FP using the built-in software. A color-coded CT map was generated automatically from a 12 × 9 mm volume scan with the inner and outer boundaries selected as the Bruch's membrane (BM) and choroidoscleral interface, respectively. Two experienced retinal specialists (Y.K. and J.O.) assessed the inner and outer segmentation lines. If necessary, the senior specialist (J.O.) manually corrected any segmentation errors.

After the CT map obtained, we also used a 6 × 6-mm SuperPixel-200 grid comprising 30 × 30 cube-pixels for objective CT measurements (Fig. 1a–c). Each cube-pixel displayed an average CT within an area of 200 × 200 μm^2^ and its coordinates. For the measurements of SCT and GCT, the SuperPixel-200 grid was centred on the fovea in the 12 × 9-mm SS-OCT scans, with manual repositioning, if necessary. The SCT value was determined automatically at a cube-pixel located on the foveal centre (Fig. 1a). The greatest CT (GCT) was also measured at the location with the highest CT value among several cube-pixels, which was located in the warm-coloured area on the color-coded CT map (Fig. 1b). The coordinates of the GCT point on the SuperPixel-200 grid were recorded for later use in determining the location of GCT point on *en face* image. The NPCT measurement point was determined using a 3.4-mm diameter circular grid for retinal nerve fibre layer analysis and measured at the point 1.7 mm nasal to the optic disc centre (Fig. 1d). NPCT was also determined using the SuperPixel-200 grid after repositioning its centre to the optic disc centre (Fig. 1c). Regional differences in CT were calculated as the ratio of CT between the two landmark locations.

### Determination of the PED morphology

The PED was evaluated by two authors (Y.K. and J.O.) to ascertain the presence, location, and extent of PED. Complicated PED was defined as PED associated with persistent SRF or CNV. Persistent SRF was determined when the presence of SRF was documented on OCT for at least 6 months without treatment such as laser photocoagulation, photodynamic therapy, or anti-VEGF injection. CNV was determined using OCT angiography, FA, and ICGA.

**Figure 2:**
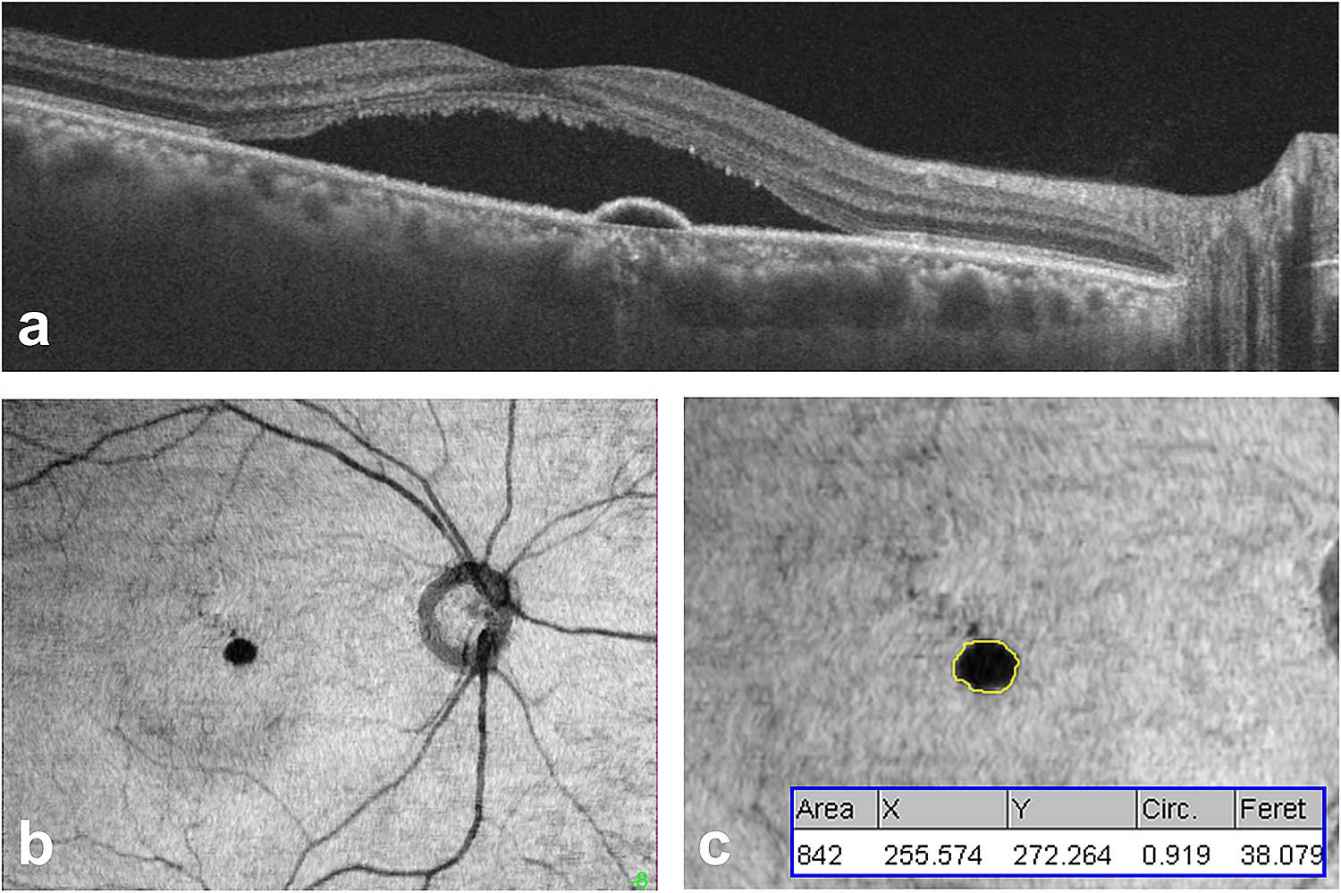
Measurement of pigment epithelial detachment (PED) on en face images of swept-source optical coherence tomography (SS-OCT). (**a**) A B-scan image of SS-OCT in a 49-year-old man with central serous chorioretinopathy shows a serous retinal detachment with subretinal fluid accumulation and serous PED in the macula. (**b**) An en face image derived from the 12 × 9-mm volume scan shows a circular hyporeflective area corresponding to the serous PED on B-scan. The slab of the en face images for PED measurement were obtained by volume flattening with reference to the Bruch’s membrane (BM). A 10.4-μm thick BM-based slab was selected at a depth position of 20.8 μm above the BM. (**c**) After tracing the boundary of the PED using the freehand tool of ImageJ software, the area, greatest linear dimension, circularity, and centroid of the PED were measured (blue box). The X and Y in the blue box represent the x and y coordinates of the PED centroid, respectively.

PED morphology was determined on *en face* images derived from the 12 × 9-mm volume scan using the built-in software (Fig. 2). The *en face* images were obtained by flattening with reference to the BM (Fig. 2b). Using the modified technique described earlier^15,20^, we selected a BM-based, 10.4-μm-thick slab, positioned 20.8 μm above the BM with considering RPE and BM thicknesses of 11–14 μm and 2–6 μm^24,25^, respectively. *En face* images were imported into ImageJ software (http://imagej.nih.gov/ij/). The PED area was determined using the freehand tool to trace the boundary of the PED (Fig. 2c, yellow line). The area, greatest linear dimension, and circularity were analysed (Fig. 2c, blue box). The centroid of the PED was automatically determined with the x- and y- coordinates. When there were two or more PEDs, the largest active PED was selected for analysis.

### Determination of the PED location

**Figure 3:**
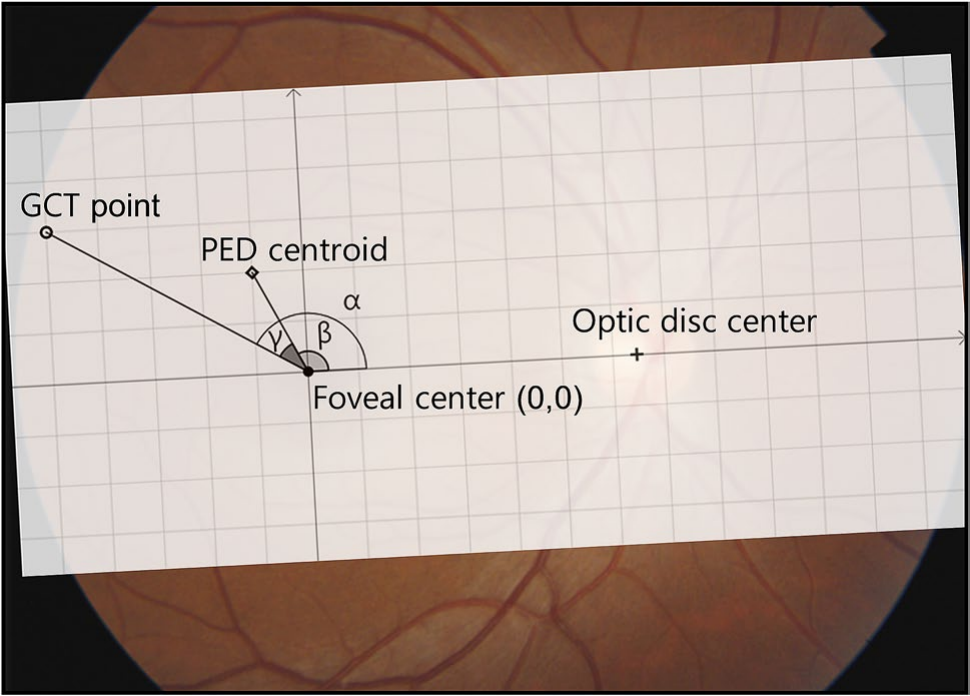
Determination of distance and angle between landmarks. For the measuring the angle, we took the foveal centre as the origin of the x- and y-axes and an imaginary line connecting the foveal centre and optic disc centre as the x-axis. Angle (α) formed by the greatest choroidal thickness (GCT) point, foveal centre, and optic disc centre. Angle (β) formed by the pigment epithelial detachment (PED) centroid, foveal centre, and optic disc centre. Angle (γ) formed by PED centroid, the foveal centre, and GCT point, was obtained as an acute angle. The angle of a landmark from the x-axis was calculated counterclockwise in the right eyes and clockwise in the left eyes. *GCT* greatest choroidal thickness, *PED* pigment epithelial detachment.

The PED location was determined as the relative location of the PED centroid to the other landmarks representing the retinal and choroidal geometries on *en face* SS-OCT images (Figs. 1, 3). To determine the PED location, we employed three reference landmarks: the foveal centre, optic disc centre, and the GCT point on *en face* images. Using the built-in software, the foveal centre and the optic disc centre were determined by observing FP and vertical/horizontal B-scans, and they were marked on the en-face image (Fig. 1). The x- and y-coordinates of GCT point on the SuperPixel-200 grid were previously determined during GCT measurement. They were used to locate the GCT point on *en face* image.

The distance or angle between landmarks was calculated after importing the marked *en face* SS-OCT images into ImageJ (Fig. 3). We used the x–y coordinates to represent each landmark. The coordinates of the PED centroid were determined using ImageJ software during the PED morphology analysis described above. The coordinates for the other three landmarks were determined using ImageJ by measuring the coordinates of the intersection of two perpendicular lines marked on the *en face* images. The distance between two landmarks were calculated using Pythagorean theorem (Fig. 3). The angles formed by the three landmarks were also calculated trigonometrically on the Cartesian coordinate plane. For the measuring the angle, we took the foveal centre as the origin of the x- and y-axes and an imaginary line connecting the foveal centre and optic disc centre as the x-axis. The angle of a landmark from the x-axis was calculated counterclockwise in the right eyes and clockwise in the left eyes. The angle, made up of a three-point PED centroid, the foveal centre, and GCT point, was obtained as an acute angle.

### Statistical analysis

All statistical analyses were performed using SPSS software (version 20.0; IBM Corp., Armonk, NY, USA). Baseline characteristics were compared using the independent t-test for continuous variables and the chi-square test or Fisher’s exact test for categorical variables. Linear correlations were analysed using Pearson’s correlation. To adjust for regional differences in CT or distance between landmarks, analyses of covariance (ANCOVA) were also performed, and the estimated marginal means of parameters were calculated and compared between groups. Results were considered statistically significant at *P* values < 0.05.

### Ethics declarations

This study was performed in line with the principles of the Declaration of Helsinki and approved by the Institutional Review Board of Korea University Anam Hospital, Seoul, Korea (IRB number: 2021AN0469), and adhered to the tenets of the Declaration of Helsinki.

### Consent to participate

This retrospective study involves no more than minimal risk to subjects and the IRB of Korea University Hospital approved our request to waive of informed consent.

## Results

### Characteristics of patients and PED

A total of 98 eyes of 98 patients with CSC were included in this study. The mean age was 51.6 ± 10.6 years (Table 1). The PED was observed in all CSC eyes, and 19 (19.4%) of them had multiple PEDs. The greatest linear dimension and circularity of the PED were correlated with NPCT (*r* = 0.207, *P* = 0.041; *r* = -0.219, *P* = 0.030). The area of the PED was correlated with the ratio of SCT to NPCT (*r* = -0.200, *P* = 0.049).

**Table 1:** Characteristics of included subjects.

Variables	Value
Number	98
Age, years	51.6 ± 10.6
**Sex, n (%)**	
Male	72 (73.5)
Female	26 (26.5)
**PED characteristics**	
Single PED, n (%)	79 (80.6)
Multiple PED, n (%)	19 (19.4)
Area, μm^2^	910,969 ± 1,959,569
Longest diameter, μm	1153 ± 1042
Circularity	0.71 ± 0.15
Complicated with persistent SRF	
Present, n (%)	46 (46.9)
Absent, n (%)	52 (53.1)
Complicated with CNV	
Present, n (%)	20 (20.4)
Absent, n (%)	78 (79.6)
**Choroidal thickness, μm**	
SCT	373 ± 107
NPCT	218 ± 84
GCT	456 ± 104
**Regional difference of CT**	
Ratio of SCT to NPCT	1.86 ± 0.64
Ratio of GCT to NPCT	2.30 ± 0.75
Ratio of GCT to SCT	1.26 ± 0.21
**Distance between landmarks**, **μm**	
From the foveal centre to the PED centroid	1091 ± 903
From the foveal centre to the GCT point	1785 ± 1202
Between PED centroid and the GCT point	1866 ± 1275
**Angle between landmarks**,°	
Formed by the PED centroid, foveal centre, and optic disc centre, counterclockwise	179.2 ± 122.9
Formed by the GCT point, foveal centre, and optic disc centre, counterclockwise	164.1 ± 95.6
Formed by the PED centroid, foveal centre, and GCT point	108.1 ± 93.4

Data are expressed as mean ± standard deviation.

*PED* pigment epithelial detachment, *SRF* subretinal fluid, *CNV* choroidal neovascularisation, *SCT* subfoveal choroidal thickness, *NPCT* nasal peripapillary choroidal thickness, *GCT* greatest choroidal thickness, *CT* choroidal thickness.

### Geometry of choroid

The mean SCT was 373 ± 10 μm. The GCT point was located outside the foveal centre in all the cases. The mean distance from the foveal centre to the GCT point (FC-GCT distance) was 1785 ± 1202 μm. The mean FC-GCT distance or mean angle formed by the GCT point, foveal centre, and optic disc centre were not correlated with age. There were no differences between males and females. The mean FC-GCT distance was correlated with SCT (*r* = -0.381, *P* < 0.001) and the ratio of GCT to SCT (*r* = 0.453, *P* < 0.001). However, it was not correlated with GCT, the ratio of SCT to NPCT, or the ratio of GCT to NPCT.

### Location of PED centroid

The centroid of the PED was not located in the foveal centre or GCT point. The distance from the foveal centre to the PED centroid (FC-PEDc distance) (1091 ± 903 μm) was smaller than the FC-GCT distance (1785 ± 1202 μm) (*P* < 0.001). The centroid of the PED was closer to the foveal centre than the GCT point (1866 ± 1275 μm) (*P* < 0.001). The mean angle formed by the PED centroid, foveal centre, and optic disc centre was 179.2 ± 122.9°. It was not different from angle formed by the GCT point, foveal centre, and optic disc centre (164.1 ± 95.6°) (*P* = 0.297). The mean angle formed by the PED centroid, foveal centre, and GCT point was 108.1 ± 93.4°.

### Factors related to the location of the PED centroid

In a total of 98 patients, the FC-PEDc distance was not correlated with age (see Supplementary Table S1 online). There was no difference between the sexes. The FC-PEDc distance was correlated with the ratio of GCT to SCT (*r* = 0.278, *P* = 0.006). However, it did not correlate with other CT parameters. The FC-PEDc distance was correlated with the FC-GCT distance (*r* = 0.371, *P* < 0.001) and with the distance between the PED centroid and GCT point (PEDc-GCT distance) (*r* = 0.361, *P* < 0.001). The PEDc-GCT distance was correlated with SCT and the ratio of GCT to SCT (*r* = -0.296, *P* = 0.003; *r* = 0.358, *P* < 0.001, respectively).

The angle formed by the PED centroid, foveal centre, and optic disc centre or the angle formed by the PED centroid, foveal centre, and GCT point were not correlated with age. There were no differences between male and female. The angle formed by the PED centroid, foveal centre, and GCT point was correlated with the distance between the PED centroid and GCT point (*r* = 0.405, *P* < 0.001).

### Comparison of PED location between eyes with and without complicated PED

**Figure 4:**
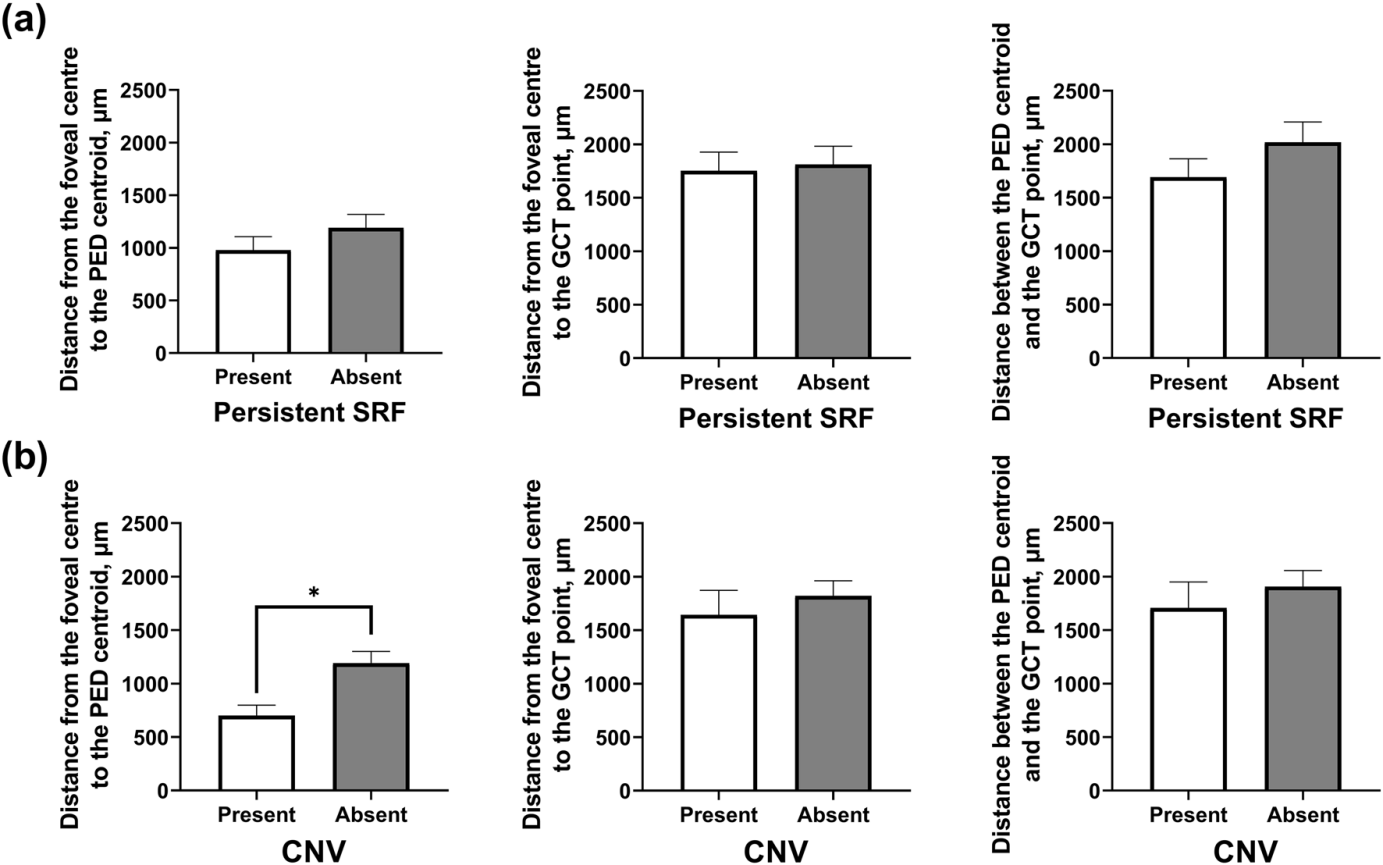
Comparison of the distances between geometric landmarks in eyes with versus those without complicated PED by persistent SRF (**a**) and choroidal neovascularisation (**b**). *PED* pigment epithelial detachment, *SRF* subretinal fluid, *CNV* choroidal neovascularisation.

Of the 98 eyes, 46 (46.9%) eyes were complicated with persistent SRF, and 52 (53.1%) eyes were not. The mean area (1,505,049 ± 2,697,298 μm^2^) and the greatest linear dimension (1529 ± 1318 μm) of the PED in eyes with persistent SRF were greater than those (385,437 ± 532,866 μm^2^ and 821 ± 542 μm) in eyes without persistent SRF (*P* = 0.008 and *P* = 0.001, respectively) (see Supplementary Table S2 online). However, the circularity of PED in eyes with persistent SRF (0.67 ± 0.15) was lower than that in eyes without persistent SRF (0.75 ± 0.12; *P* = 0.010). The mean FC-PEDc distance did not differ between eyes with and without persistent SRF (Fig. 4a). The mean angle formed by the PED centroid, foveal centre, and GCT point in eyes with persistent SRF was not different from those without.

Of the 98 eyes, 20 (20.4%) eyes were complicated with CNV, and 78 (79.6%) eyes were not. PED morphology and CT profile differed between eyes with and without CNV. The mean FC-PEDc distance was shorter in eyes with CNV (700 ± 439 μm) than in those without CNV (1191 ± 964 μm) (*P* = 0.001; 95% CI, -784.3 to -198.4) (Fig. 4b). Mean angle formed by the PED centroid, foveal centre, and GCT point was smaller in the PED group with CNV (75.0 ± 62.5°) than in those without (116.6 ± 98.4°) (*P* = 0.024; 95% CI, -77.5 to -5.6).

Of the 46 eyes with PED complicated with persistent SRF, 20 (43.5%) were also complicated with CNV and were classified into the CNV group, while 26 (56.5%) eyes without CNV were classified into the non-CNV group (see Supplementary Table S2 online). The mean FC-PEDc distance was smaller in the CNV group (700 ± 440 μm) than in the non-CNV group (1194 ± 1049 μm) (*P* = 0.037; 95% CI, -956.5 to -31.5). The angle formed by the PED centroid, foveal centre, and GCT point did not differ between groups.

### Analyses of covariance for factors related to the location of the PED centroid

ANCOVA including the FC-GCT distance, ratio of GCT to SCT, and the PED group with and without CNV showed that the FC-PEDc distance was significantly affected by the FC-GCT distance (*P* = 0.009) and PED group with and without CNV (*P* = 0.020) (Table 2). The estimated marginal means of the FC-PEDc distance in the PED group with the CNV group (700 ± 185 μm) was smaller than that in the non-CNV group (1191 ± 93 μm) (*P* = 0.020) (Table 3).

**Table 2:** Analysis of covariance for factors related to location of the centroid of the pigment epithelium detachment.

Source of variation	Sum of square	d.f	Mean of squares	*F* value	*P* value
Ratio of GCT to SCT	1,829,382.40	1	1,829,382.40	2.721	0.102
Distance from the GCT point to the foveal centre	4,845,272.60	1	4,845,272.60	7.206	0.009
PED group with and without CNV	3,750,408.30	1	3,750,408.30	5.578	0.020

*GCT* greatest choroidal thickness, *SCT* subfoveal choroidal thickness, *PED* pigment epithelium detachment, *CNV* choroidal neovascularisation.

**Table 3:** Estimated marginal means with 95% confidence intervals of distance from the PED centroid to the foveal centre between the PED group complicated with and without CNV.

Variables	Groups	Estimated marginal mean	SE	95% CI (lower–upper)	*P* value
Distance from PED centroid to foveal centre, μm	PED with CNV	700	185	333–1068	0.020*
	PED without CNV	1191	93	1006–1376	

Data were evaluated at covariates appearing in the model: ratio of the greatest choroidal thickness to subfoveal choroidal thickness = 1.26, distance from the greatest choroidal thickness point to foveal centre = 1785 μm.

*SE* standard error, *CI* confidence interval, *PED* pigment epithelial detachment, *CNV* choroidal neovascularisation.

## Discussion

In CSC, the term “central” refers to the central or macular centre of the posterior pole; therefore, in CSC, PED has been hypothesized to occur around the fovea^1,2,26^. In this study, we showed that the PED centroid is located around the GCT point. This is consistent with previous findings^27,28^ that CSC develop in association with choroidal thickening. However, the GCT points were not located in the subfoveal region, but were more frequent in the superotemporal region. In contrast to earlier studies^29,30^ that used a limited number of OCT line scans, recent studies^22,23,31^ using volumetric CT maps from SS-OCT revealed that the choroid was thickest in the superotemporal region, even in healthy subjects. In the present study, PED was closer to the foveal centre than the GCT point. This implies that choroidal thickening is not the only factor contributing to the location of PED. Recent studies^6,7,32,33^ using ICGA and ultra-wide imaging have shown that choroidal vascular insufficiency or stasis is associated with the pathogenesis of CSC. Dansingani et al.^14^ suggested that the abrupt narrowing of large choroidal vessels in the macula is responsible for the development of CSC. Recently, the presence of intervortex venous anastomosis in patients with CSC has been suggested^34,35^. Intervortex venous anastomoses were located around the fovea and optic disc. These large choroidal vessels were hypothesized to be induced by choroidal venous outflow dysfunction. Increased hydrostatic pressure caused by choroidal congestion and choriocapillaris hyperpermeability, as well as RPE dysfunction caused by the choriocapillaris insufficiency overlying the dilated choroidal vessels, could contribute to the development of PED. These findings are consistent with our finding that the PED centroid was close to the foveal centre. This supports the suggestion that the development of PED does not necessarily begin at the point of greatest CT.

In this study, the location of the PED centroid did not depend on age or sex, suggesting that the location of PED may be more influenced by local factors than demographic factors. To account for ocular factors related to the development of PED, the location of the PED centroid was compared with the CT profile. The FC-PEDc distance was correlated with the FC-GCT and PEDc-GCT distances. The PEDc-GCT distance was correlated with SCT and the ratio of GCT to SCT. This finding supports the suggestion that changes in choroidal geometry are responsible for initiating the RPE changes^36^. However, the FC-PEDc distance was not different between eyes with and without persistent SRF. This may suggest that the PED location cannot be helpful in predicting the chronicity of SRF in CSC.

CNV is known to be present in more cases of CSC than expected^17–19,37^. Possibility of an accompanying CNV should be considered, especially in patients with flat and irregular PED^15,17–19,37^. In this study, we found that PED complicated with CNV was closer to the foveal centre than those without CNV. Our findings may help suspect PED, which requires further studies to determine the likelihood of developing CNV. After controlling for the effect of choroidal geometry including the GCT to SCT ratio and the FC-GCT distance, the PED location was closer to the FC in eyes with CNV than in those without CNV. This means that CT is not the only factor contributing to the development of a complicated PED with CNV. It is not clear why PED complicated with CNV is located closer to the foveal centre than those without CNV. RPE in the subfoveal region may be more susceptible to hypoxia or degenerative changes than the RPE outside the subfoveal region^38^. This may have contributed to the development of CNV in PEDs located near the foveal centre. In addition, regardless of the GCT location, the distribution of dilated large choroidal vessels in the macula could have influenced the PED location complicated by CNV. Dilated large choroidal vessels could have induced chronic damage to the subfoveal RPE, leading to the development of CNV^16,34,35,39^.

FA and ICGA were used to determine the leak point of CSC. After the introduction of OCT, it was found that the location of the leakage was related to the PED location on the OCT image. In 2005, van Velthoven et al.^4^ evaluated the extent of CSC using *en face* OCT. Recently, Lumbroso et al.^20^ showed that the aetiology of PED can be assessed using *en face* OCT, and Ferrara et al.^15^ also used *en face* SS-OCT images to present a topographical association between choroidal changes and PED. However, in most studies, the PED location was determined without quantitative measurements. Assuming that the centre of the PED is the origin of the PED, we attempted to determine the location of the PED by measuring its centre. However, in most cases, the centre of the PED could not be determined because the PED was not circular. Instead, we chose the centroid as the centre of the PED. To measure the relative location of the PED centroid with respect to retinal geography, we determined the location of the foveal centre and optic disc in the *en face* OCT images. We also selected additional landmarks, such as GCT points for choroidal geography, considering that variations in CT are associated with the development of PED in CSC. These landmarks allowed us to quantitatively determine the location of PED. We found that the mean angle formed by the PED centroid, foveal centre, and GCT point was about 108°, indicating that PED occurred on the same side as choroidal thickening. We also found that the angle was not different between eyes with and without persistent SRF, suggesting that the location of the PED is not an issue that causes chronic CSC. This method can help determine the relative location of the pathological changes associated with variations in CT.

This study has some limitations owing to its retrospective nature and small sample size. Although age and sex were included as demographic factors in the current study, systemic factors such as hypertension and diabetes that could affect CT were not included in this study. More studies with a larger number of participants that evaluate systemic factors affecting CT may be required. The area or length was measured from the OCT images. However, this method can be affected by peripheral distortions and refractive errors. Another limitation of our study is that we did not examine the relationship between visual acuity and geometric landmarks such as the PED location and GCT point. In this study, we assumed that the centroid represented the location of the PED. However, the actual growth of the PED could have not begun from the centroid. This may have influenced the results of the present study. The size or shape of the PED differed between the eyes with and without complicated PED. However, the location of the PED centroid was not affected by the size or shape of the PED, suggesting that the centroid is a stable measurement for the PED.

In summary, we quantitatively measured CT and the location of symptomatic PED in patients with CSC. First, the PED centroid was closer to the foveal centre than the GCT point. Second, both choroidal geometry and the presence of CNV were related to the PED location. Third, the location of PED showed a tendency to get closer to the centre in the case of CNV, although the PED location was farther away from the foveal centre as the GCT point was farther from the foveal centre. In conclusion, this result suggests that the development of complicated PED can be related to both choroidal vascular abnormalities and RPE insufficiency in the fovea.

## Supplementary Information

Supplementary Tables.

## Data Availability

The datasets generated during and/or analysed during the current study are available from the corresponding author on reasonable request.
