# Supplementary material for: Factors related to the location of pigment epithelial detachment in central serous chorioretinopathy
Source: Sci Rep. 2022 Mar 16;12:4507. doi: 10.1038/s41598-022-08550-0 (PMC8927148; doi:10.1038/s41598-022-08550-0)
Supplement: Supplementary file 1 — Supplementary Tables. [file 41598_2022_8550_MOESM1_ESM.pdf]

Factors related to the location of pigment epithelial detachment in central serous chorioretinopathy

Young Ho Kim, MD, PhD, Edward Kang, MD, Jaeryung Oh, MD, PhD

Department of Ophthalmology, Korea University College of Medicine, Seoul, Korea

A List of Supplementary Tables

- **Supplementary Table 1.** Correlation between location of pigment epithelium detachment and other parameters
- **Supplementary Table 2.** Comparison between pigment epithelial detachment with and without complication
- **Supplementary Table 3.** Subgroup analysis for 46 eyes with pigment epithelium detachment complicated with persistent subretinal fluid

Supplementary Table S1. Correlation between location of pigment epithelium detachment and other parameters

Variables	Distance from the foveal centre to the PED centroid		Distance from the PED centroid to the GCT point		Angle formed by the PED centroid, foveal centre, and optic disc centre		Angle formed by the PED centroid, foveal centre, and GCT point	
	r	*P* value	*r*	*P* value	*r*	*P* value	*r*	*P* value
Age	0.022	0.827	-0.071	0.489	0.083	0.418	-0.172	0.091
Area of PED	-0.097	0.343	-0.061	0.549	-0.026	0.799	-0.077	0.449
Largest diameter of PED	-0.044	0.667	-0.066	0.517	-0.065	0.528	-0.143	0.159
Circularity of PED	-0.044	0.668	0.020	0.847	0.088	0.391	0.148	0.146
SCT	-0.084	0.413	-0.296	0.003*	-0.033	0.748	0.078	0.447
GCT	0.060	0.555	-0.145	0.154	-0.018	0.858	0.060	0.557
NPCT	0.061	0.553	-0.097	0.343	0.208	0.040*	0.051	0.615
Ratio of SCT to NPCT	-0.072	0.483	-0.098	0.335	-0.305	0.002*	0.055	0.589
Ratio of GCT to NPCT	0.053	0.602	0.066	0.518	-0.305	0.002*	0.016	0.873
Ratio of GCT to SCT	0.278	0.006*	0.358	< 0.001*	-0.002	0.982	-0.085	0.405
Distance from the foveal centre to the PED centroid	-	-	0.361	< 0.001*	-0.275	0.006*	0.023	0.821
Distance from the foveal centre to the GCT point	0.371	< 0.001*	0.779	< 0.001*	-0.122	0.231	0.056	0.581
Distance between the PED centroid and the GCT point	0.361	< 0.001*	-	-	-0.104	0.310	0.405	< 0.001*

**P* value < 0.05 by Pearson's correlation.

PED, pigment epithelium detachment; SCT, subfoveal choroidal thickness; GCT, greatest choroidal thickness; NPCT, nasal peripapillary choroidal thickness.

Supplementary Table S2. Comparison between pigment epithelial detachment with and without complication

	Persistent SRF			CNV		
	Present	Absent	*P* value	Present	Absent	*P* value
Number of eyes	46	52		20	78	
Age, years	56.2 ± 9.0	47.6 ± 9.3	<0.001*	60.1 ± 8.8	49.5 ± 9.2	< 0.001*
Sex, male to female, n	36 : 10	36 : 16	0.364	15 : 5	57 : 21	> 0.999
PED morphology						
Area, μm^2	1505049 ± 2697298	385437 ± 532866	0.008*	2870720 ± 3670645	408469 ± 509193	0.007*
Longest diameter, µm	1529 ± 1318	821 ± 542	0.001*	2335 ± 1582	850 ± 543	0.001*
Circularity	0.67 ± 0.15	0.75 ± 0.12	0.010*	0.62 ± 0.18	0.73 ± 0.13	0.013*
Choroidal thickness, µm						
SCT	340 ± 96	402 ± 108	0.004*	324 ± 107	386 ± 104	0.020*
NPCT	223 ± 91	214 ± 79	0.580	233 ± 95	214 ± 82	0.371
GCT	423 ± 93	485 ± 105	0.002*	407 ± 93	468 ± 103	0.018*
Regional difference of CT						
Ratio of SCT to NPCT	1.66 ± 0.55	2.03 ± 0.66	0.004*	1.45 ± 0.29	1.96 ± 0.66	< 0.001*
Ratio of GCT to NPCT	2.11 ± 0.75	2.47 ± 0.72	0.016*	1.87 ± 0.44	2.41 ± 0.78	< 0.001*
Ratio of GCT to SCT	1.28 ± 0.21	1.24 ± 0.21	0.384	1.30 ± 0.17	1.25 ± 0.22	0.344
Distance between landmarks, µm						
From the foveal centre to the PED centroid	979 ± 868	1190 ± 929	0.251	700 ± 439	1191 ± 964	0.001*
From the foveal centre to the GCT point	1754 ± 1182	1813 ± 1230	0.810	1644 ± 1020	1821 ± 1248	0.557
Between the PED centroid and the GCT point	1692 ± 1163	2020 ± 1360	0.205	1707 ± 1083	1906 ± 1323	0.535
Angle between landmarks, °						
Formed by the PED centroid, foveal centre, and optic disc centre, counterclockwise	168.9 ± 119.7	188.4 ± 126.1	0.436	184.5 ± 111.9	177.9 ± 126.2	0.830
Formed by the GCT point, foveal centre, and optic disc centre, counterclockwise	156.2 ± 100.3	171.1 ± 91.7	0.444	143.7 ± 90.2	169.4 ± 96.8	0.286
Formed by the PED centroid, foveal centre, and GCT point	97.5 ± 90.6	117.5 ± 95.7	0.291	75.0 ± 62.5	116.6 ± 98.4	0.024*

Note: Data are expressed as mean ± standard deviation.

*P value < 0.05 by independent t-test or chi-square test.

Abbreviations: SRF, subretinal fluid; CNV, choroidal neovascularisation; PED, pigment epithelial detachment; SCT, subfoveal choroidal thickness; NPCT, nasal peripapillary choroidal thickness; GCT, the greatest choroidal thickness; CT, choroidal thickness

Supplementary Table S3. Subgroup analysis for 46 eyes with pigment epithelium detachment complicated with persistent subretinal fluid

	CNV group	Non-CNV group	*P* value
Number	20	26	
Age, years	60.1 ± 8.8	53.1 ± 8.1	0.008*
Sex, male to female, n	15 : 5	21 : 5	0.453
PED morphology			
Area, μm2	2,870,720 ± 3,670,645	454,532 ± 464,775	0.009*
Longest diameter, μm	2,335 ± 1582	908 ± 551	0.001*
Circularity	0.62 ± 0.18	0.71 ± 0.11	0.058
Choroidal thickness, μm			
SCT	324 ± 107	353 ± 87	0.309
NPCT	233 ± 96	215 ± 89	0.516
GCT	407 ± 93	435 ± 93	0.325
Regional difference of CT			
Ratio of SCT to NPCT	1.45 ± 0.29	1.83 ± 0.64	0.010*
Ratio of GCT to NPCT	1.87 ± 0.44	2.28 ± 0.88	0.047*
Ratio of GCT to SCT	1.30 ± 0.17	1.26 ± 0.88	0.570
Distance between landmarks, μm			
From the foveal centre to the PED centroid	700 ± 440	1,194 ± 1,049	0.037*
From the foveal centre to the GCT point	1,644 ± 1,020	1,839 ± 1,307	0.584
Between the PED centroid and the GCT point	1,707 ± 1,083	1,680 ± 1,242	0.939
Angel between landmarks, °			
Formed by the PED centroid, foveal centre, and optic disc centre, counterclockwise	184.5 ± 111.9	156.8 ± 126.2	0.443
Formed by the GCT point, foveal centre, and optic disc centre, counterclockwise	143.7 ± 90.2	165.9 ± 108.2	0.464
Formed by the PED centroid, foveal centre, and GCT point	75.0 ± 62.5	114.7 ± 105.3	0.119

* P value < 0.05 by independent t-test or Fisher’s exact test.

CNV, choroidal neovascularisation; PED, pigment epithelium detachment; SCT, subfoveal choroidal thickness; NPCT, nasal peripapillary choroidal thickness; GCT, greatest choroidal thickness; CT, choroidal thickness.
